# Supplementary material for: Ultrafast energy relaxation dynamics of amide I vibrations coupled with protein-bound water molecules
Source: Nat Commun. 2019 Mar 1;10:1010. doi: 10.1038/s41467-019-08899-3 (PMC6397197; doi:10.1038/s41467-019-08899-3)
Supplement: Supplementary file 1 — Supplementary Information [file 41467_2019_8899_MOESM1_ESM.docx]

Supplementary Information

Ultrafast Energy Relaxation Dynamics of Amide I Vibrations Coupled with Protein-Bound Water Molecules

Junjun Tan, Jiahui Zhang, Chuanzhao Li, Yi Luo* & Shuji Ye*

Supplementary Figure 1. Schematic representation of the experimental geometry.

Supplementary Figure 2. The IIV-SFG cross-correlation traces of water bending mode.

Supplementary Figure 3. Relaxation time of amide I mode measured in D_2_O and organic solvents is plotted against the amide I frequency.

Supplementary Figure 4. The three-level vibrational model.

Supplementary Figure 5. The ‘pump on’ (red curve) and ‘pump off’ (black curve) spectra at different delay times.

Supplementary Figure 6. The intensity decay of the amide I band of different peptides with ν_pump_ = ν_probe_ = 1660 cm^-1^.

Supplementary Figure 7. The intensity decay of the water bending mode at CaF_2_/water interface.

Supplementary Figure 8. The intensity decay of the water bending mode at DPPG bilayer/water interface.

Supplementary Figure 9. The relationship between HDX ratio / 1.15ps component ratio and amide I frequency.

Supplementary Table 1. The peptide concentration used for the interaction between different peptides and lipid bilayers

Supplementary Table 2. The relaxation time of amide I mode investigated in D_2_O or organic solvents

Supplementary Table 3. Fitting amplitude ratio($\chi_{5h\text{ HDX}}^{(2)}/\chi_{before\text{ HDX}}^{(2)}$) of the ssp SFG spectra in the N-H region after 5h HDX and before HDX, and the amide I frequency.

Supplementary Note 1. The procedures of prism-cleaning and lipid monolayer/bilayer preparation.

Supplementary Note 2. Time-resolved sum frequency generation (TR-SFG) system and experiments

Supplementary Note 3. The vibrational relaxation of amide I mode in D_2_O and organic solvents

Supplementary Note 4. The hydrogen-deuterium exchange (HDX) of the amide proton

Supplementary Note 5. The explicit expressions for the intensity decay of the amide I band

Supplementary Note 6.The ‘pump on’ and ‘pump off’ spectra at some typical delay times

Supplementary Note 7. Controlled experiments on the excitation of water bending mode at CaF_2_/H_2_O interface and DPPG bilayer/ H_2_O interface

Supplementary Note 8. Analyzing using two energy relaxation processes


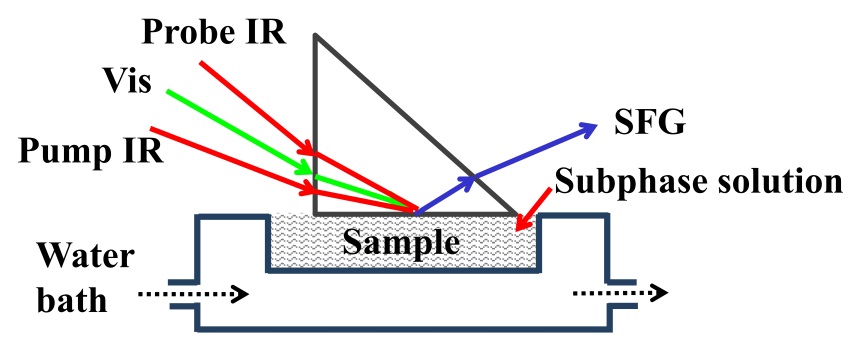


**Supplementary Figure 1. Schematic representation of the experimental geometry.**

**Supplementary Figure 2.** **The IIV-SFG cross-correlation traces of water bending mode at different peptide-inserted DPPG bilayer/water interface under current experimental geometry.** **Upper panel** : pure DPPG bilayer/water interface; **middle panel** : KALP23-inserted DPPG bilayer/water interface; **lower panel** : melittin-inserted DPPG bilayer/water interface.

**Supplementary Figure 3**. **Relaxation time of amide I mode measured in D_2_O(solid circle, black) and organic solvents(open circle,blue) is plotted against the amide I frequency.**


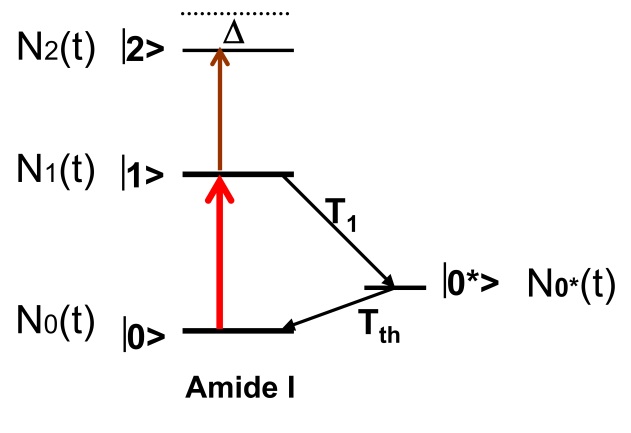


**Supplementary Figure 4**. **The three-level vibrational model was used to analyze the vibrational dynamics of amide I mode.**

**Supplementary** **Figure 5**. **The ‘pump on’ (red curve) and ‘pump off’ (black curve) spectra at different delay times.** **a** Melittin; **b** LKα14; **c** MP; **d** BM2; **e** AM2 at pH =4; **f** AM2 at pH =6.2; **g** AM2 at pH =8.5; **h** KALP23 (H_2_O); **i** KALP23 (D_2_O).

**Supplementary** **Figure 6**. **The intensity decay of the amide I band of different peptides with ν_pump_ = ν_probe_ = 1660 cm^-1^.** The red lines are the fitting curves. We fitted the data for delay time at t ≤ 0.1 ps using the function of 1-A_0_ exp (t/T_0_) and fitted the data for delay time at t ≥ 0.1 ps using Supplementary Eq. S18 for the peptides of Melittin, LKα14, MP and AM2. For the peptides of BM2 and KALP23, the data for delay time at t ≥ 0.1 ps was fitted using Supplementary Eq. S16 because of relatively large bleaching.

**Supplementary Figure 7**. **The intensity decay of the water bending mode at CaF_2_/water interface.** **a** The static ssp SFG spectrum at CaF_2_/water interface: the red solid dot represents the ‘pump on’ spectrum at delay time of 0.2 ps, the black dot represents the ‘pump off’ spectrum, the blue triangle is the corresponding differential transient SFG spectra (I_pump on_- I_pump off_). **b** The intensity decay of water bending mode with ν_pump_ = 1660cm^-1^ and ν_probe_ = 1660cm^-1^. **c** The differential transient SFG spectra (I_pump on_- I_pump off_) in the region of water bending mode. To avoid the spectra to overlap together, the spectra were offset with a certain value.

**Supplementary Figure 8**. **The intensity decay of the water bending mode at DPPG bilayer/water interface. a** The static ssp SFG spectrum at CaF_2_ -supporting DPPG bilayer/water interface: the red solid dot represents the ‘pump on’ spectrum at delay time of 0.2ps, the black dot represents the ‘pump off’ spectrum, the blue triangle is the corresponding differential transient SFG spectra (I_pump on_- I_pump off_). **b** The intensity decay of water bending mode with ν_pump_ = 1660 cm^-1^ and ν_probe_ = 1660 cm^-1^. **c** The differential transient SFG spectra (I_pump on_- I_pump off_) in the region of water bending mode. To avoid the spectra to overlap together, the spectra were offset with a certain value.

**Supplementary Figure 9. The relationship between HDX ratio / 1.15ps component ratio and amide I frequency.** Red: the ratio of the component of 1.15 ps component is plotted against the amide I frequency; Black: fitting amplitude ratio ($\chi_{5h\text{ HDX}}^{(2)}/\chi_{before\text{ HDX}}^{(2)}$) of the ssp SFG spectra in the N-H region after 5h HDX and before HDX against the amide I frequency.

**Supplementary Table 1.** The peptide concentration used for the interaction between different peptides and lipid bilayers

| Peptide | Peptide concentration | Peptide amount (μL) | Lipid bilayer |
| --- | --- | --- | --- |
| Melittin | 2 mg/mL | 2 | DPPG/DPPG |
| LKα-14 | 2 mg/mL | 10 | DMPG/DMPG |
| MP | 2 mg/mL | 5 | d-DMPC/d-DMPC |
| BM2 | 2 mg/mL | 10 | DMPG/DMPG |
| AM2 | 2 mg/mL | 20 | POPG/POPG |
| KALP23 | 1 mg/mL | 5 | DPPG/DPPG |

**Supplementary Table 2**. The relaxation time of amide I mode investigated in D_2_O or organic solvents

| Peptide group or protein | Amide I frequency | Solvent | Relaxation time(ps) | Ref. |
| --- | --- | --- | --- | --- |
| Glycine-L-Alanine-Methyl amide dipeptide | 1674.7 ± 0.5cm^-1^  1646.3 ± 0.5cm^-1^ | D_2_O | 1.2±0.2 | 14 |
| Isoniazid | 1644 cm^-1^ | D_2_O | 1.2±0.1 | 15 |
| Small de novo cyclic pentapeptide (cyclo-Mamb-Abu-ArgGly-Asp) | 1652 cm^-1^ | D_2_O | 1.2±0.1 | 16 |
| Apamin, scyllatoxin, and bovine pancreatic trypsin inhibitor (BPTI) | 1645 cm^-1^ | D_2_O | 1.2±0.1 | 17 |
| Myoglobin | 1653 cm^-1^ | D_2_O | 1.3±0.2 | 18 |
| Myoglobin | 1655 cm^-1^ | D_2_O | 1.2-1.3 | 19 |
| KALP23 | 1663 cm^-1^ | D_2_O | 1.15±0.05 | Current study |
| N‑Propionyl‑D‑glucosamine | 1628.0 cm^-1^ | D_2_O | 1.03±0.03 | 20 |
| N‑Propionyl‑D‑glucosamine | 1661 cm^-1^ | DMSO | 1.217±0.019 | 20 |
| *N*-acetyl-L-prolinamide | 1682 cm^-1^ | CDCl_3_ | 1.1±0.1 | 21 |
| acetylproline-NH2 | 1693 cm^-1^ | CH_2_Cl_2_ | 1.2±0.1 | 22 |

**Supplementary** **Table 3**. Fitting amplitude ratio($\chi_{5h\text{ HDX}}^{(2)}/\chi_{before\text{ HDX}}^{(2)}$) of the ssp SFG spectra in the N-H region after 5h HDX and before HDX, and the amide I frequency

| peptides | Melittin | LKα14 | BM2 | AM2 | KALP23 |
| --- | --- | --- | --- | --- | --- |
| Peak center of Amide I (cm^-1^) | 1651.0 | 1652.1 | 1657.6 | 1662.4 | 1664.8 |
| $\chi_{5h\text{ HDX}}^{(2)}/\chi_{before\text{ HDX}}^{(2)}$ | 0.12 | 0.25 | 0.55 | 0.91 | 0.97 |

**Supplementary Note 1. The procedures of prism-cleaning and lipid monolayer/bilayer preparation.**

CaF_2_ prisms were thoroughly cleaned using a procedure with several steps: They were first soaked in toluene for at least 24 h and then sonicated in soap detergent solution for 0.5 h. After that, they were rinsed with deionized (DI) water before soaking in methanol for 10 min. All of the prisms were then rinsed thoroughly with an ample amount of DI water and cleaned inside Harrick plasma chamber for 10 min immediately before depositing lipid molecules on them. Substrates were tested using SFG and no signal from contamination was detected.

Single lipid bilayers were prepared on CaF_2_ substrates using Langmuir-Blodgett and Langmuir-Schaefer (LB/LS) methods with a KSV mini trough LB system. The lipid monolayer was deposited on CaF_2_ prism surface to produce the monolayer/air surface. The bilayer was prepared by contacting the CaF_2_ prism’s monolayer-coated right-angle face with the monolayer of lipid at 35 mN/m surface pressure at the water surface. The bilayers were immersed in pure water inside a 2 mL reservoir throughout the entire experiments, and a small amount of water could be added to the reservoir to compensate for evaporation when needed for long time scale experiments. The detailed procedure was similar to previous reports^1,2^.

The membrane-bound peptides were prepared by interacting peptides with CaF_2_ prism-supported lipid bilayer at 24°C (see **Supplementary Figure 1**). A magnetic micro stirrer was used to ensure a homogeneous concentration of the peptide molecules in the subphase below the bilayer. The peptide without inserting into lipid bilayer was carefully removed by replacing the DI water or acid solution in the subphase. The lipid and peptide concentration used for the interaction between different peptides and lipid bilayer were given in Supplementary Table 1.

**Supplementary Note 2. Time-resolved sum frequency generation (TR-SFG) system and experiments**

Details regarding SFG theories and instruments have been reported previously^3-8^. The TR-SFG system has been introduced in details in references 9 and 10. In this study, the bandwidth of the pump IR and probe IR pulses is about 170 cm^-1^ (FWHM)^10^.

**The determination of time-zero.**For the SFG probe, the time-zero was determined by monitoring the infrared-visible sum-frequency generation signals from the amide I band of the peptides as a function of the delay between the IR probe and the visible pulses. For the IR pump- SFG probe, the time-zero and instrument response were determined according to previous reports^10-12^, i.e., by monitoring the third-order cross-correlation of infrared-infrared visible (IIV) sum-frequency signals from the water bending mode as a function of the delay between the IR pump and IR probe pulses. The IIV-SFG cross-correlation traces of water bending mode at peptide-inserted DPPG bilayers/ water interface are given in Supplementary Figure 2. The full width at half maximum is about 285±5 fs. According to the coherence length, the IIV-SFG only probes the bulk molecules in the interfacial distance below ~80 nm. Therefore, the IIV SFG signals from the water bending mode can be used to determine the time-zero and instrument response of the IR pump- SFG probe process.

**Spectra acquisition and data processing.** A LABVIEW program was used to control the delay time between the IR pump and the IR probe, as well as to switch the pump IR on and off by controlling TOPAS shutter. For every delay time, a system consisted of chopper and galvanometric scanner was used to minimize the influence of laser fluctuations on the pump-on and pump-off spectra. Each spectrum has a collect time of 1.0 s (500 laser shots) with 100 times accumulation. It takes 117.5 s (including the instrumental response time) to collect one spectrum. It takes about two hour to collect all of the transient spectra with the delay time ranging from -3 ps to 8 ps.

The obtained time-resolved IR pump- SFG probe spectra were measured as a function of the delay time (Δt) between the IR pump and the SFG probe pair. The intensity decay of the vibrational modes we present in this study is given by integrating the spectra in the frequency from ω_ν_ - Γ_ν_ to ω_ν_ + Γ_ν_ at each delay time. All the IR pump-SFG probe transients presented here were collected under sspp (s-polarized SFG output, s-polarized visible input, p-polarized infrared probe, p-polarized infrared pump) polarization combinations. The energy profiles of the IR pulses were used to normalize the SFG spectra.

We fitted the normalized spectra using a standard procedure, Supplementary Eq. 1.

$I_{SFG}\propto\left|\chi_{NR}^{(2)}+\sum_{\nu} \frac{A_{\nu}}{\omega_{IR}-\omega_{\nu}+i\Gamma_{\nu}}\right|^{2}$ (1)

where $A_{\nu}$, $\omega_{\nu}$, and $\Gamma_{\nu}$ are the strength, resonant frequency, and damping coefficient of the vibrational mode(ν), respectively. $\chi_{NR}^{(2)}$ is the non-resonant background. $A_{\nu}$ could be either positive or negative depending on the phase of the vibrational mode^3-8^.

**The method to avoid the laser induced heat.** Because the pump process is not interface specific, the water near the interface may absorb the infrared pump pulse. In order to reduce such effect, a home-made water bath (see Supplementary Figure 1) was used to maintain the sample temperature at 24°C. The subphase of the bilayer was surrounded by the flowing water in water bath, which can effectively avoid the heating caused by the laser.

**Supplementary Note 3. The vibrational relaxation of amide I mode in D_2_O and organic solvents**

The vibrational relaxation of the amide I modes for model compounds of the peptide group and some globular peptides or proteins has been investigated by ultrafast infrared spectroscopy such as 2D IR and time-resolved IR pump-IR probe methods. Because the frequency of amide I mode is strongly overlapped with the H_2_O bending mode absorption, the IR experiments were generally carried out in D_2_O or organic solvents^13-23^. The relaxation time of preivous studies is summarized in Supplementary Table 2. It is found that vibrational relaxation of the amide I mode all occurs in ca. 1.2 ps (Supplementary Figure 3)^14-22^. In the light of the fact, the population relaxation of the amide I mode is considered as an intrinsic property of the peptide group itself and depends very little on the surrounding environment of the peptide bond or side chains^13-23^.

**Supplementary Note 4. The hydrogen-deuterium exchange (HDX) of the amide proton**

Previous studies indicated that the residues flanked at the lipid/water interfaces can undergo hydrogen-deuterium exchange(HDX) of the amide proton rapidly following the sample exposure to deuterium, but the part lied in the core of lipid bilayer does not exchange in the 3-4 days^10,24,25^. Therefore, we can determine the relative amount of peptide bonds that are exposed to H_2_O by measuring the HDX ratio of the amide proton. We first prepared the membrane-bound peptides at the subphase of H_2_O. After the interactions between peptides and lipid bilayers reach equilibrium, we measured the SFG spectra in the amide A band and amide I bands. And then we replaced the subphase solution of lipid bilayer by D_2_O carefully. After 5 h to allow HDX to take place, we collected SFG spectra of the peptides at lipid bilayer/D_2_O interface again. Figure 1A shows the ssp SFG spectra of the amide A band at lipid membrane interface. The spectra in black curves are measured at lipid bilayer/H_2_O interface while the one in red curved are measured at lipid bilayer/D_2_O interface after 5-h HDX experiments. Figure 1B shows the ssp amide I SFG spectra of the peptides in lipid membrane/H_2_O interface. To qualitatively determine the 5h HDX ratio and the amide I frequency change of these peptides, we fitted the spectra using a standard procedure, Supplementary Eq. S1. The fitting results are given in Supplementary Table 3.

**Supplementary Note 5. The explicit expressions for the intensity decay of the amide I band**

Because SFG intensity depends on the square of the surface population density of the vibrational state^3,4^, the pump IR pulse at 1660 cm^-1^ excites the amide I mode from the ground (ν = 0) to its first vibrational state (ν = 1) and then reduce the SFG intensity. Three-level vibrational model (Supplementary Figure 4) has been used to extract the vibrational lifetime (T_1_) and thermalization time constant (T_th_) of the vibrational dynamics in many condensed matters, including water^11,26,27^ and peptides^22,28-30^. In this model, the amide I modes are excited from ν=0 to ν=1 state and it takes T_1_ time to relax to a “hot” ground state (ν=0*), and then the intensity of the amide I band gradually recovers. The decay of the ν=0* state with a vibrational cooling time of T_th_ leads to a full thermalization of the system. The detailed description of three-level model is given in our previous studies ^11,26,27^.

Because the anharmonicity of the C=O vibration is small and it cannot separate the SFG signal generated from the ground and excited states, therefore, the signal from the excited state will contribute to the signal of $I_{\nu_{0}}.$ According to Supplementary Eq.S1, the nonlinear susceptibility after excitation $\chi_{pump on}^{\left( 2 \right)}(t)$ can be given by

$\chi_{pump on}^{\left( 2 \right)}\left( t \right)=A_{NR}+\frac{A_{01}\left( N_{0}\left( t \right)-N_{1}\left( t \right) \right)}{\omega_{IR}-\omega_{01}+i\Gamma_{01}}+\frac{A_{12}{(N}_{1}\left( t \right)-N_{2}\left( t \right))}{\omega_{IR}-\omega_{12}+i\Gamma_{12}}+\frac{A_{01}*N_{0}*\left( t \right)}{\omega_{IR}-\omega_{01}+i\Gamma_{01}}$ (2)

where N_x_(t) is the population of the x state of the amide I group at time t. A_ij_, ω_ij_, Γ_ij_ are the amplitude, frequency, and damping constant, respectively, for ν=i → ν=j transition. A_01_^*^ and N_0_^*^ are the amplitude and population of the amide I mode in the “hot” ground states. Here, we assume all the resonant terms have the same phase sign with respect to the non-resonant background. We can assume that after the initial IR excitation and equilibration, we only have significant population in the |0⟩ and |1⟩ states. Therefore, we can set N_2_(t) =0. At any point in time before or after excitation, the total population satisfies $N_{0}\left( t \right)+N_{1}\left( t \right)+N_{0}*\left( t \right)=N_{0}$, and then Supplementary Eq.S2 can be written as

$\chi_{pump on}^{\left( 2 \right)}\left( t \right)=A_{NR}+\frac{A_{01}\left( N_{0}-{2N}_{1}\left( t \right) \right)}{{\omega_{IR}-\omega}_{01}+i\Gamma_{01}}+\frac{A_{12}N_{1}\left( t \right)}{{\omega_{IR}-\omega}_{12}+i\Gamma_{12}}+\frac{r{A_{01}N}_{0}*\left( t \right)}{\omega_{IR}-\omega_{01}+i\Gamma_{01}}$ (3)

Where *r*=(*A*_01_^*^-*A*_01_)/*A*_01_.

The intensity ratio beween pump on and pump off can be written as

$I_{pump on}/I_{pump off}\propto\left| \chi_{pump on}^{\left( 2 \right)}\left( t \right)/\chi_{pump off}^{\left( 2 \right)} \right|^{2}=\left| (A_{NR}+\frac{A_{01}\left( N_{0}-{2N}_{1}\left( t \right) \right)}{{\omega_{IR}-\omega}_{01}+i\Gamma_{01}}+\frac{A_{12}N_{1}\left( t \right)}{{\omega_{IR}-\omega}_{12}+i\Gamma_{12}}+\frac{r{A_{01}N}_{0}*\left( t \right)}{\omega_{IR}-\omega_{01}+i\Gamma_{01}})/(A_{NR}+\frac{A_{01}N_{0}}{{\omega_{IR}-\omega}_{01}+i\Gamma_{01}}) \right|^{2}$ (4)

Because the nonresonant part *A_NR_* is very small and negligible compared to the resonant part, therefore, we have following relationship,

$\frac{\chi_{pump on}^{\left( 2 \right)}\left( t \right)}{\chi_{pump off}^{\left( 2 \right)}}=1-{2n}_{1}\left( t \right)+\frac{{n_{1}\left( t \right)A}_{12}\left( {\omega_{IR}-\omega}_{01}+i\Gamma_{01} \right)}{A_{01}\left( {\omega_{IR}-\omega}_{12}+i\Gamma_{12} \right)}+rn_{0}*\left( t \right)$ (5)

where $n_{1}\left( t \right)=N_{1}\left( t \right)/N_{0}$, $n_{0*}\left( t \right)=N_{0*}\left( t \right)/N_{0}$.

According to the harmonic approximation, both the Raman transition moment and the IR dipole moment are proportional to (j+1)^1/2 31^. Therefore, the molecular hyperpolarizability of the excited state (*A*_12_) is twice of the ground state(*A*_01_). In addition, we can define that ω_01_=ω_12_+Δ and Γ_12_ = Γ_01_. Here, Δ is anharmonic shift. With these definitions, Supplementary Eq. S5 can be further simplified as

(6)

where .

According to the previous analyses of the ultrafast dynamics of condensed matter^11,26,27^, the dynamics of the population change of each state in Supplementary Figure 4 can be described by using the following differential equations with the assumptions: 1) the pump pulse follows a δ function at the time t_0_; 2) The population of the excited state at t= t_0_ is $(1-\sqrt{S_{0}})/2k$, here S_0_ is the SFG signal at t=t_0_. t_0_ corresponds to the time of the minimum of the SFG signal; 3) there is no population in the intermediate state and “hot” ground state at t= t_0_; 4) when the delay time is infinite, there is no population in the excited and intermediate. With these boundary conditions

$\frac{dn_{1}\left( t \right)}{dt}=-\frac{n_{1}\left( t \right)}{T_{1}}$ (7)

$\frac{{dn}_{0*}(t)}{dt}=\frac{n_{1}(t)}{T_{1}}-\frac{n_{0*}(t)}{T_{th}}$ (8)

$\frac{{dn}_{0}(t)}{dt}=\frac{n_{0*}(t)}{T_{th}}$ (9)

(10)

$n_{0*}\left( t_{0} \right)=0$ (11)

Solving the equations in Supplementary Eqs.S7-S8 gives,

(12)

(13)

Substituting Supplementary Eqs. S10- S13 into Supplementary Eq.S6 gives,

$\frac{\chi_{pump on}^{\left( 2 \right)}\left( t \right)}{\chi_{pump off}^{\left( 2 \right)}}=1-{2kn}_{1}\left( t \right)+rn_{0*}\left( t \right)=1-(1-\sqrt{S_{0}}{)e}^{-\frac{t-t_{0}}{T_{1}}}+\frac{\frac{r\left( 1-\sqrt{S_{0}} \right)}{2k}}{\frac{T_{1}}{T_{th}}-1}\left( e^{-\frac{t-t_{0}}{T_{1}}}-e^{-\frac{t-t_{0}}{T_{th}}} \right)$

$=1-(1-\sqrt{S_{0}}{-\Delta S)e}^{-\frac{t-t_{0}}{T_{1}}}-\Delta Se^{-\frac{t-t_{0}}{T_{th}}}$ (14)

where $\Delta S=\frac{\frac{r\left( 1-\sqrt{S_{0}} \right)}{2k}}{\frac{T_{1}}{T_{th}}-1}$.

The intensity ratio beween pump on and pump off can be further simplified as

$$\frac{I_{pump on}}{I_{pump off}}\propto\left| \frac{\chi_{pump on}^{\left( 2 \right)}\left( t \right)}{\chi_{pump off}^{\left( 2 \right)}} \right|^{2}=\left( 1-(1-\sqrt{S_{0}}{-\Delta S)e}^{-\frac{t-t_{0}}{T_{1}}}-\Delta Se^{-\frac{t-t_{0}}{T_{th}}} \right)^{2}=$$

$\frac{I_{pump on}}{I_{pump off}}=1-2(1-\sqrt{S_{0}}-\Delta S{)e}^{-\frac{t-t_{0}}{T_{1}}}-2\Delta Se^{-\frac{t-t_{0}}{T_{th}}}+(1-\sqrt{S_{0}}{{-\Delta S)}^{2}e}^{-\frac{2\left( t-t_{0} \right)}{T_{1}}}+2\Delta S(1-\sqrt{S_{0}}-\Delta S{)e}^{-(t-t_{0})/T_{1}}e^{-\frac{t-t_{0}}{T_{th}}}+(\Delta S)^{2}e^{-2(t-t_{0})/T_{th}}$ (15)

It is worth noting in our study that ΔS is very small. Supplementary Eq.S15 can be approximated to be,

$\frac{I_{pump on}}{I_{pump off}}=1-2(1-\sqrt{S_{0}}-\Delta S{)e}^{-\frac{t-t_{0}}{T_{1}}}-2\Delta Se^{-\frac{t-t_{0}}{T_{th}}}+(1-\sqrt{S_{0}}-\Delta S{)^{2}e}^{-2(t-t_{0})/T_{1}}$ (16)

Accordingly, if $(1-\sqrt{S_{0}}-\Delta S$) is very small, Supplementary Eq.S16 can be approximated to be

$\frac{I_{pump on}}{I_{pump off}}=1-2(1-\sqrt{S_{0}}-\Delta S{)e}^{-\frac{t-t_{0}}{T_{1}}}-2\Delta Se^{-\frac{t-t_{0}}{T_{th}}}$ (17)

At this case, the decay in the SFG intensity approximately equals to the population decay time. When $(1-\sqrt{S_{0}}$) and $\Delta S$ are very small, $2(1-\sqrt{S_{0}})\approx1-S_{0}$. And then Supplementary Eq.S17 can be changed into Supplementary Eq.S18.

$\frac{I_{pump on}}{I_{pump off}}=1-(1-S_{0}{-2\Delta S)e}^{-\frac{t-t_{0}}{T_{1}}}-2\Delta Se^{-\frac{t-t_{0}}{T_{th}}}$ (18)

**Supplementary Note 6.The ‘pump on’ and ‘pump off’ spectra at some typical delay times**

**Supplementary** **Figure 5** shows the ‘pump off’ (red curve) and ‘pump on’ (black curve) spectra at different delay times. It can be seen that the bandwidth of the ‘pump on’ spectra of amide I modes at different delay times is similar to the one of the ‘pump off’ spectra. In general, if the excitation of the water bending mode affects the dynamics of amide I mode, the bandwidth will become broader. Therefore, the influence of the excitation of water bending mode can be excluded. The intensity ratio of I_pump on_/ I_pump off_ is obtained (Supplementary Figure 6) by integrating the spectra in the frequency from ω_ν_ - Γ_ν_ to ω_ν_ + Γ_ν_ at each delay time. Here, the ω_ν_ and Γ_ν_ are peak center and damping coefficient of the Amide I mode given by Supplementary Eq. S1, respectively. For melittin, the intensity ratio of I_pump on_/ I_pump off_ is obtained by integrating the spectra in the frequency from 1640 cm^-1^ to 1670 cm^-1^ at each delay time.

**Supplementary Note 7. Controlled experiments on the excitation of water bending mode at CaF_2_/H_2_O interface and DPPG bilayer/ H_2_O interface**

We have performed controlled experiments to investigate the influence of the excitation of water bending mode on the amide I modes. It is found that the bleaching in frequency range of 1600-1700 cm^-1^ is negligible when we pump the water bending mode at CaF_2_/H_2_O interface and DPPG bilayer/ H_2_O interface. Therefore, the effect of the excitation of water bending mode on the vibrational dynamics of amide I is very small, at least beyond the experimental errors.

**Supplementary Note 8. Analyzing using two energy relaxation processes**

According to Figure 1A, the amide I groups can actually be considered as two components: the one coupled to water and the other not coupled to water. Therefore, the intensity decay of the amide I band shown in Supplementary Figure 6 could also be analyzed using two energy relaxation processes: one for the amide modes coupled to water with relaxation time of 0.4 ps (a value approximate to the lifetime of water bending modes ^32,33^) and one for the amide modes not coupled to water with relaxation time of 1.15 ps (the lifetime of amide I mode in D_2_O). The ratio of the component of 1.15 ps is plotted in Supplementary Figure 9. It is evident that the ratio of the component of 1.15 ps in H_2_O environment linearly correlates with the amide I frequency and matches well with the exposure amount of membrane-bound peptides to the water determined by HDX method (Figure 1D). This result suggests that the relaxation time measurements combining with the site-specific labeling technique developed by Zanni et al.^34,35^ will offer a unique and effective optical marker to determine the hydrophobicity of specific sites.

**Supplementary References**

1. Zhang, J. H., Yang, W. L., Tan, J. J. & Ye, S. J. In situ examination of a charged amino acid-induced structural change in lipid bilayers by sum frequency generation vibrational spectroscopy. *Phys. Chem. Chem. Phys.* **20**, 5657-5665 (2018).

Chen, X., Wang, J., Kristalyn, C. B. & Chen, Z. Real-time structural investigation of a lipid bilayer during its interaction with melittin using sum frequency generation vibrational spectroscopy. *Biophys. J.* **93**, 866-875 (2007).

1. Shen, Y. R. *The Principles of Nonlinear Optics* (Wiley New York, 1984).
2. Lambert, A. G., Davies, P. B. & Neivandt, D. J. Implementing the theory of sum frequency generation vibrational spectroscopy: a tutorial review. *Appl. Spectrosc. Rev.* **40**, 103-145 (2005).
3. Yan, E. C. Y., Fu, L., Wang, Z. G. & Liu, W. Biological macromolecules at interfaces probed by chiral vibrational sum frequency generation spectroscopy. *Chem. Rev.* **114**, 8471-8498 (2014).
4. Ding, B., Jasensky, J., Li, Y. & Chen, Z. Engineering and characterization of peptides and proteins at surfaces and interfaces: a case study in surface-sensitive vibrational spectroscopy. *Acc. Chem. Res.* **49**, 1149-1157 (2016).
5. Schach, D. K. et al. Reversible activation of a cell-penetrating peptide in a membrane environment. *J. Am. Chem. Soc.* **137,** 12199-12202 (2015).
6. Wang, H. F., Gan, W., Lu, R., Rao, Y. & Wu, B. H. Quantitative spectral and orientational analysis in surface sum frequency generation vibrational spectroscopy (SFG-VS). *Int. Rev. Phys. Chem.* **24**, 191-256 (2005).

Tan, J. J., Luo, Y. & Ye, S. J. A highly sensitive femtosecond time-resolved sum frequency generation vibrational spectroscopy system with simultaneous measurement of multiple polarization combinations. *Chin. J. Chem. Phys.* **30**, 671-677 (2017).

1. Tan, J. J., Zhang, B. X., Luo, Y. & Ye, S. J. Ultrafast vibrational dynamics of membrane-bound peptides at the lipid bilayer/water interface. *Angew. Chem. Int. Ed.* **56**, 12977-12981 (2017).
2. Ghosh, A., Smits, M., Bredenbeck, J. & Bonn, M. Membrane-bound water is energetically decoupled from nearby bulk water: an ultrafast surface-specific investigation. *J. Am. Chem. Soc.* **129**, 9608-9609 (2007).
3. Bonn, M. et al. Structural inhomogeneity of interfacial water at lipid monolayers revealed by surface-specific vibrational pump−probe spectroscopy. *J. Am. Chem. Soc.* **132**, 14971-14978 (2010).
4. Ghosh, A. & Hochstrasser, R. M. A peptide’s perspective of water dynamics. *Chem. Phys.* **390**, 1-13 (2011).
5. Candelaresi, M. et al. Conformational analysis of Gly–Ala–NHMe in D_2_O and DMSO solutions: a two-dimensional infrared spectroscopy study. *J. Phys. Chem. B* **117**, 14226-14237 (2013).
6. Shaw, D. J. et al. Multidimensional infrared spectroscopy reveals the vibrational and solvation dynamics of isoniazid. *J. Chem. Phys.* **142**, 212401 (2015).
7. Hamm, P., Lim, M., DeGrado, W. F. & Hochstrasser, R. M. Pump/probe self heterodyned 2D spectroscopy of vibrational transitions of a small globular peptide. *J. Chem. Phys.* **112**, 1907-1916 (2000).
8. Hamm, P., Lim, M. & Hochstrasser, R. M. Structure of the amide I band of peptides measured by femtosecond nonlinear-infrared spectroscopy. *J. Phys. Chem. B* **102,** 6123-6138 (1998).
9. Peterson, K. A., Rella, C. W., Engholm, J. R. & Schwettman, H. A. Ultrafast vibrational dynamics of the myoglobin amide I band. *J. Phys. Chem. B* **103**, 557-561 (1999).
10. Le Caer, S. et al. The effect of myoglobin crowding on the dynamics of water: an infrared study. *Phys. Chem. Chem. Phys.* **16**, 22841-22852 (2014).
11. Han, C., Zhao, J., Yang, F. & Wang, J. P. Structural dynamics of N-propionyl-_D_-glucosamine probed by infrared spectroscopies and ab initio computations. *J. Phys. Chem. A* **117**, 6105-6115 (2013).
12. Sul, S., Karaiskaj, D., Jiang, Y. & Ge, N. H. Conformations of N-acetyl-_L_-prolinamide by two-dimensional infrared spectroscopy. *J. Phys. Chem. B* **110**, 19891 -19905 (2006).
13. Rubtsov, I. V. & Hochstrasser, R. M. Vibrational dynamics, mode coupling, and structural constraints for acetylproline-NH_2_. *J. Phys. Chem. B* **106**, 9165-9171 (2002).
14. Mukherjee, P., Kass, I., Arkin, I. T. & Zanni, M. T. Picosecond dynamics of a membrane protein revealed by 2D IR. *Proc. Natl. Acad. Sci. USA* **103**, 3528-3533 (2006).
15. Dunkelberger, E. B., Woys, A. M. & Zanni, M. T. 2D IR cross peaks reveal hydrogen–deuterium exchange with single residue specificity. *J. Phys. Chem. B* **117**, 15297-15305 (2013).
16. Demmers, J. A., Haverkamp, J., Heck, A. J., Koeppe, R. E. & Killian, J. A. Electrospray ionization mass spectrometry as a tool to analyze hydrogen/deuterium exchange kinetics of transmembrane peptides in lipid bilayers. *Proc. Natl. Acad. Sci. USA* **97**, 3189-3194 (2000).
17. Nienhuys, H. K., Woutersen, S., van Santen, R. A. & Bakker, H. J. Mechanism for vibrational relaxation in water investigated by femtosecond infrared spectroscopy. *J. Chem. Phys.* **111**, 1494-1500 (1999).
18. Lock, A.J., Woutersen, S. & Bakker, H.J. Ultrafast energy equilibration in hydrogen-bonded liquids. *J. Phys. Chem. A* **105**, 1238-1243(2001).

DeCamp, M. F., DeFlores, L., McCracken, J. M., Tokmakoff, A., Kwac, K. & Cho, M. Amide I vibrational dynamics of N-methylacetamide in polar solvents: The role of electrostatic interactions. *J. Phys. Chem. B* **109**, 11016-11026 (2005).

Rubtsov, I.V., Wang, J.P. & Hochstrasser, R.M. Dual-frequency 2D-IR spectroscopy heterodyned photon echo of the peptide bond. *Proc. Natl. Acad. Sci. USA* **100**, 5601-5606(2003).

1. Rubtsov, I.V., Wang, J. P. & Hochstrasser, R. M. Vibrational coupling between amide-I and amide-A modes revealed by femtosecond two color infrared spectroscopy. *J. Phys. Chem. A* **107**, 3384-3396 (2003).
2. Guyotsionnest, P. Two-phonon bound state for the hydrogen vibration on the H/Si(111) surface. *Phys. Rev. Lett.* **67**, 2323-2326 (1991).
3. Piatkowski, L. & Bakker, H. J. Vibrational dynamics of the bending mode of water interacting with ions. *J. Chem. Phys.* **135**, 214509 (2011)
4. Larsen, O. F.A. & Woutersen, S. Vibrational relaxation of the H_2_O bending mode in liquid water. *J. Chem. Phys.* **121**, 12143-12145 (2004).
5. Ding, B., Panahi, A., Ho, H.J., Laaser, J.E., Brooks III, C.L., Zanni, M.T. & Chen, Z. Probing site-specific structural information of peptides at model membrane interface in situ. *J. Am. Chem. Soc.* **137**, 10190-10198(2015).
6. Dunkelberger, E.B., Woys, A.M. & Zanni, M.T. 2D IR cross peaks reveal hydrogen−deuterium exchange with single residue specificity. *J. Phys. Chem. B* **117**, 15297-15305 (2013).
